# Endophthalmitis rates following intravitreal injection of aflibercept using a prefilled syringe – a multicenter study

**DOI:** 10.1007/s00417-026-07285-2

**Published:** 2026-05-29

**Authors:** Daniel David, Dinah Zur, Amit Biran, Sharon Braudo, Koby Brosh, Elad Moisseiev, Shiri Shulman, Yoreh Barak, Rita Ehrlich, Shalev Fried, Alon Peled, David Hauser, Tareq Jaouni, Eran Pras, Hagay Hayat, Gabriel Katz

**Affiliations:** 1 Department of Ophthalmology, Sheba Medical Center, Faculty of Medical and Health Sciences, Goldschleger Eye Institute, Tel Aviv University, Tel Aviv, Israel; 2 Division of Ophthalmology, Tel Aviv Medical Center, Faculty of Medical and Health Sciences, Tel Aviv University, Tel Aviv, Israel; 3 Department of Ophthalmology, Hadassah Medical Center, Faculty of Medicine, The Hebrew University of Jerusalem, Jerusalem, Israel; 4 Department of Ophthalmology, Shaare Zedek Medical Center, Faculty of medicine, Hebrew University of Jerusalem, Jerusalem, Israel; 5 Department of Ophthalmology, Faculty of Medical and Health Sciences, Meir Medical Center, Tel Aviv University, Tel Aviv, Israel; 6 Ophthalmology Institute, Assuta Medical Centers, Tel Aviv, Israel; 7 Department of Ophthalmology, Rambam Health Care Campus, Haifa, Israel; 8 Wolfson Medical Center, Holon, Israel; 9 Department of Ophthalmology, Assaf-Harofeh Medical Center, Zerifin, Israel, Affiliated to the Faculty of Medical and Health Sciences, Tel Aviv University, Tel Aviv, Israel; 10 Department of Ophthalmology, Faculty of Health Sciences, Barzilai Medical Center, Ben-Gurion University, Ashkelon, Be’er Sheba, Israel; 11 Rappaport Faculty of Medicine, Technion-Israel Institute of Technology, Haifa, Israel; 12 Faculty of Health Sciences, Ben-Gurion University of the Negev, Beersheba, Israel; 13 Gray Faculty of Medical and Health Sciences, Tel Aviv University, Tel Aviv, Israel

**Keywords:** Endophthalmitis, Anti-vascular endothelial growth factor (VEGF), Pre-filled syringe, Intra-vitreal injection, Aflibercept

## Abstract

**Purpose:**

To evaluate endophthalmitis rates following intravitreal injection of aflibercept, comparing between vial preparation and prefilled syringe use.

**Methods:**

We retrospectively collected data from ten ophthalmology departments in Israel. The number of intravitreal anti-VEGF injections administered in years 2022 and 2023 was recorded. Endophthalmitis rates in 2022 (vial use) and in 2023 (prefilled syringe use) were compared. Clinical details of endophthalmitis cases were reviewed.

**Results:**

In 2022, out of 119,904 intravitreal injections, 31,991 (27%) were aflibercept injections prepared from vials. In 2023, out of 128,306 intravitreal injections, 35,589 (28%) were aflibercept injections from prefilled syringes. Endophthalmitis rates were similar between vial preparation (1:1991) and prefilled syringe (1:2372) (*p* = 0.63). Notably, aflibercept injections exhibited significantly higher endophthalmitis rates compared to bevacizumab and ranibizumab combined, in both years (1:1999 vs. 1:5494 in 2022, and 1:2373 vs. 1:7132 in 2023, respectively; *p* < 0.01 for both).

**Conclusion:**

Despite the transit to prefilled aflibercept syringes, endophthalmitis rates following aflibercept injection did not decrease. Post-injection endophthalmitis rates were significantly higher with aflibercept compared to bevacizumab and ranibizumab, with both vial and prefilled syringe.

## Key messages

**What is known:**

- Pre-filled syringes for intravitreal bevacizumab and ranibizumab injections have improved sterility and lower rates of post-injection endophthalmitis compared to the vial ones.

- Aflibercept intravitreal injections, previously available in the vial form, are now widely administered via a pre-filled syringe.

**What is new:**

- The switch from vial to pre-filled aflibercept syringes did not result in a decrease in endophthalmitis rates.
- Regardless of syringe type, aflibercept showed higher post-injection endophthalmitis rates compared with bevacizumab and ranibizumab.

## Introduction

Intravitreal injection of an anti-vascular endothelial growth factor (anti-VEGF) agent is the primary treatment of several vision threatening retinal diseases [1–3], including neovascular age-related macular degeneration (AMD) [4], diabetic macular edema (DME) [5], and retinal vein occlusion (RVO) [6]. The three most commonly used anti-VEGF agents in the years 2022–2023 were bevacizumab (Avastin^®^; Genentech Inc., South San Francisco, CA, US), ranibizumab (Lucentis^®^; Novartis Pharmaceuticals, Basel, Switzerland) and aflibercept (Eylea^®^; Bayer HealthCare, Berlin, Germany), all delivered as injection of 0.05 mL into the vitreous cavity [7–9]. 

Initially, the conventional method of administering intravitreal injections involved manually drawing the drug from a vial, which posed certain challenges related to precision [10], dosage accuracy [11, 12], preparation time [13] and the risk of contamination [14–19]. These drawbacks have led to the introduction of pre-filled syringe (PFS) injections, where the drug is preloaded in a sterile syringe. Bacterial endophthalmitis, although a relatively rare event, is the most dreaded complication of intravitreal injections, with potentially devastating visual outcomes [20]. Observational studies comparing ranibizumab vial and PFS formulations have reported a reduction in the rates of endophthalmitis following the introduction of the PFS, supporting the hypothesis that minimizing handling steps during preparation may reduce contamination risk [21–23]. 

Aflibercept was initially introduced only as a vial formulation and has become one of the most widely used anti-VEGF agents worldwide. However, unlike ranibizumab, the impact of the PFS formulation on endophthalmitis rates in aflibercept injections remains less clearly defined. A manufacturer-based database study suggested a lower incidence of intraocular inflammation and endophthalmitis with the aflibercept PFS compared with the vial formulation [24]. Yet this report combined infectious endophthalmitis with sterile intraocular inflammation, limiting the ability to accurately estimate the risk of true bacterial endophthalmitis. Furthermore, real-world clinical data evaluating the effect of transitioning from vial to PFS formulations failed to demonstrate a statistically significant reduction in infectious endophthalmitis rates with the use of aflibercept PFS [25]. 

PFS of aflibercept were introduced in several countries only recently, including in our country at the beginning of 2023, providing a unique opportunity to compare outcomes before and after the transition from vial-based preparation to the PFS formulation. Given the widespread use of aflibercept and the limited clinical evidence regarding the effect of its PFS formulation on infectious endophthalmitis rates, further clinical data is needed to clarify whether the theoretical advantages of the PFS translate into improved safety outcomes.

In this study we aim to review and compare the rates of endophthalmitis following intravitreal aflibercept (IVT-AFL) injections using the PFS versus the vial presentation. We postulated that the endophthalmitis rates would decrease after the transition to the PFS.

## Methods

We retrospectively collected data from ophthalmology departments in ten different medical centers, all in the same country. The study was approved by the institutional review boards of all hospitals and adhered to the tenets of the Declaration of Helsinki.

The total number of intravitreal injections of anti-VEGF agents administered from January 1, 2022, to December 31, 2023, was acquired through electronic clinic records and validated with the economy units of each medical center. Injections were classified according to the anti-VEGF agent (bevacizumab, ranibizumab, or aflibercept).

### Injection procedure

All intravitreal injections were performed as office-based procedures, and the technique was similar throughout the study period. All injections were performed under topical anesthesia, in a supine position under sterile conditions. A sterile plastic drape was used to move the eyelashes away from the ocular surface, and a sterile speculum was inserted. Then, 1–2 drops of 4% povidone-iodine were applied to the conjunctiva over the injection site and left to contact for at least 60 s. Intravitreal injection was performed using the straight injection technique. Following the injection, modest pressure was applied with a sterile cotton-tipped applicator over the injection site to reduce vitreal reflux and subconjunctival hemorrhage. Residual povidone-iodine was rinsed with saline.

## Syringe types

There were two types of syringe-filling techniques: manual preparation in which the drug was drawn by the injecting physician from the commercial vial, and use of a PFS. During the study period, all injections of bevacizumab and ranibizumab were executed using PFS. Until December 31, 2022, aflibercept was prepared manually by self-drawing of the drug from a sterile vial into the syringe, performed by the injecting physician. From January 1, 2023, PFS for intravitreal aflibercept injections became commercially available throughout the country and all participating medical centers began using it instead of the vial.

### Patients and clinical data

All cases of presumed infectious endophthalmitis following intravitreal injection of anti-VEGF that were treated at the same medical center or in any of the other participating medical centers were included. The main inclusion criteria were a clinical diagnosis of endophthalmitis, made by an experienced retina specialist, deemed severe enough to necessitate intervention (pars plana vitrectomy or tap and inject). Patients presented with mild post-injection inflammatory reactions were excluded. Cases were classified as either culture-positive or culture-negative endophthalmitis based on vitreous culture results. When cultures were not obtained, or when cultures yielded no growth, the cases were categorized as infectious endophthalmitis according to clinical presentation (pain, decrease in visual acuity, hypopyon, vitritis). The diagnosis of endophthalmitis was made clinically by experienced retina specialists.

The following data were retrieved for analysis: Age, gender, injection date, anti-VEGF agent injected, agent preparation (prefilled or manually drawn), and the indication for injection. Pre-operative and post-operative clinical features were also noted and included visual acuity, lens status, type of intervention (pars plana vitrectomy or tap-injection), time to intervention and culture results. Patients suffering from endophthalmitis following other interventions were excluded, as well as patients that were injected in other medical centers that did not participate in this study, as this did not allow to calculate the incidence of the complication.

All cases were treated with either vitreous tap or vitrectomy, combined with intravitreal injection of antibiotics. Information on whether vitreous fluids were sent for cultures was extracted, and what were the isolated organisms in culture-positive cases.

The main outcome measure was the rate of endophthalmitis following intravitreal aflibercept injections using the PFS, compared to vial preparation. Secondary outcome measures were the rate of total endophthalmitis and culture-positive endophthalmitis following intravitreal aflibercept injections compared to bevacizumab and ranibizumab injections.

### Statistical analysis

Quantitative variables were described as mean and standard deviation. Categorical variables were described as absolute and relative frequencies.

Pearson’s chi-square test and Fisher’s exact test were used to compare endophthalmitis rates following aflibercept injections compared to bevacizumab and ranibizumab injections and to evaluate the change in endophthalmitis rate before and after the transition to PFS.

The overall significance level was set to an alpha of 0.05. The statistical analysis was carried out using Microsoft Excel 2017 (Microsoft Corporation, Redmond, WA).

## Results

In all 10 medical centers, a total of 119,904 intravitreal injections were given in 2022, of which 31,991 (26.7%) were aflibercept with vial preparation, compared to 35,589 (27.7%) IVT-AFL PFS injections in 2023, from a total of 128,306 injections. A sum of 76,528 (63.8%) PFS bevacizumab injections were given in 2022 and 82,310 (64.1%) in 2023, while the number of ranibizumab injections was 11,385 (9.5%) and 10,407 (8.1%) in 2022 and 2023, respectively, all as PFS (Table 1).

**Table 1:** Endophthalmitis incidence by anti-VEGF agent and year

Agent	Year	Endophthalmitis cases (*n*)	Total injections (*n*)	Ratio	Incidence per 10,000 injections
Bevacizumab	2022	14	76,528	1:5466	1.83
Bevacizumab	2023	10	82,310	1:8231	1.21
Aflibercept	2022	16	31,991	1:1999	5.00
Aflibercept	2023	15	35,589	1:2373	4.21
Ranibizumab	2022	2	11,385	1:5692	1.76
Ranibizumab	2023	3	10,407	1:3469	2.88

Overall comparison between drugs across the entire study period, χ² test of proportions, *p* < 0.001

Pairwise Fisher exact tests, Bevacizumab vs. Aflibercept *p* < 0.001, Bevacizumab vs. Ranibizumab *p* = 0.387, Aflibercept vs. Ranibizumab *p* = 0.175

In total, sixty cases of post-intravitreal injection endophthalmitis were documented in the years 2022–2023: 31 (51.6%) occurred following IVT-AFL injections, 24 (40%) after bevacizumab and 5 (8.3%) after ranibizumab injection. Due to the relatively small number of ranibizumab injections in our study, we grouped the bevacizumab and ranibizumab endophthalmitis cases together and compared them as a group to aflibercept injections. Table 2 summarizes the clinical and demographic characteristics of the study group, divided by the type of injected agent.

**Table 2:** Characteristics of endophthalmitis cases by anti-VEGF agent

Variable	Bevacizumab (*n* = 24)	Aflibercept (*n* = 31)	Ranibizumab (*n* = 5)	*P* value
Age^*^, median (IQR)	78.0 (71.8–82.5)	78.0 (72.0–84.5)	89.0 (73.0–91.0)	0.762 (Kruskal–Wallis)
Male^#^, n (%)	15 (62.5)	13 (42)	1 (20)	0.132 (χ²)
Female^#^, n (%)	9 (37.5)	18 (58)	4 (80)	
Diabetes mellitus^#^, n (%)	10 (42)	9 (29)	0 (0)	0.172 (χ²)
Indication^#^				0.015 (χ²)
AMD, n (%)	16 (67)	23 (74)	3 (60)	
DME, n (%)	6 (25)	6 (19)	0 (0)	
RVO, n (%)	1 (4)	2 (6)	0 (0)	
Other, n (%)	1 (4)	0 (0)	2 (40)	
Primary intervention^#^				0.513 (χ²)
Pars plana vitrectomy	8 (33)	16 (52)	1 (20)	
Tap & inject	15 (63)	14 (45)	4 (80)	
N/A	1 (4)	1 (3)	0 (0)	

Values are presented as median (interquartile range) for continuous variables^*^ and number (percentage) for categorical variables^#^

Continuous variables^*^ were compared using the Kruskal–Wallis test. Categorical variables^#^ were compared using the chi‑square (χ²) test or Fisher’s exact test when appropriate. A p-value < 0.05 was considered statistically significant

**Fig. 1:**
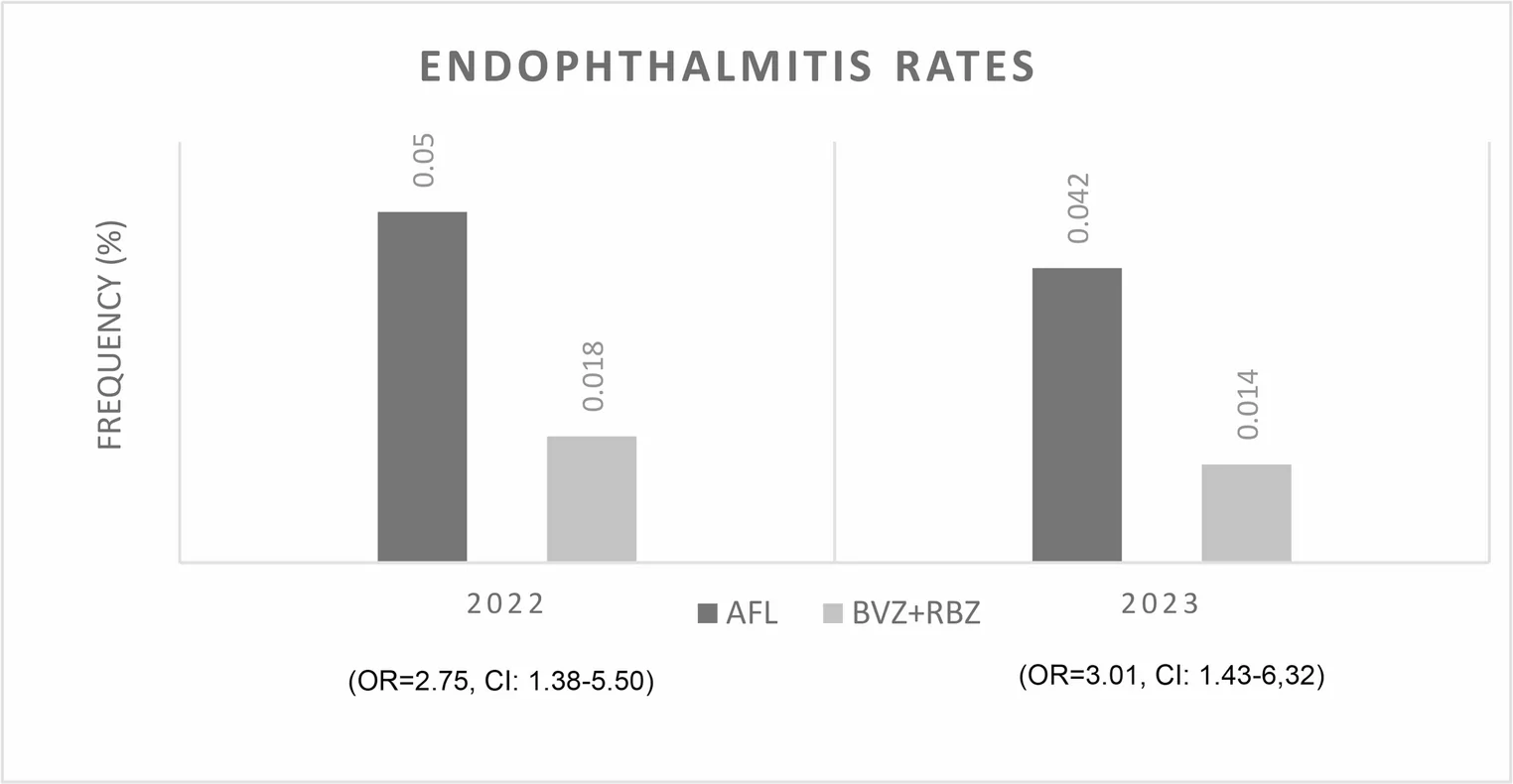
Comparison of endophthalmitis rates with aflibercept compared with bevacizumab and ranibizumab in 2022 and 2023

Rates of endophthalmitis after IVT-AFL injections (1:1999) were 2.75 times higher compared with the combined rates of bevacizumab and ranibizumab (1:5494) in 2022 (OR = 2.75, 95% CI 1.38–5.50, *p* = 0.005). In 2023, endophthalmitis rates following IVT-AFL injections were 1:2373, compared to 1:7132 following bevacizumab and ranibizumab (OR = 3.01, 95% CI 1.43–6.32, *p* = 0.005), (Fig. 1). No significant difference was noted in the endophthalmitis rates when comparing IVT-AFL vial injections in 2022 with IVT-AFL PFS injections in 2023 (OR = 1.19, 95% CI 0.59–2.40, *p* = 0.63).

*Endophthalmitis rates were significantly higher following intravitreal aflibercept injection compared with bevacizumab and ranibizumab in 2022 and 2023. AFL= aflibercept; BVZ = bevacizumab; RBZ = ranibizumab.*

**Fig. 2:**
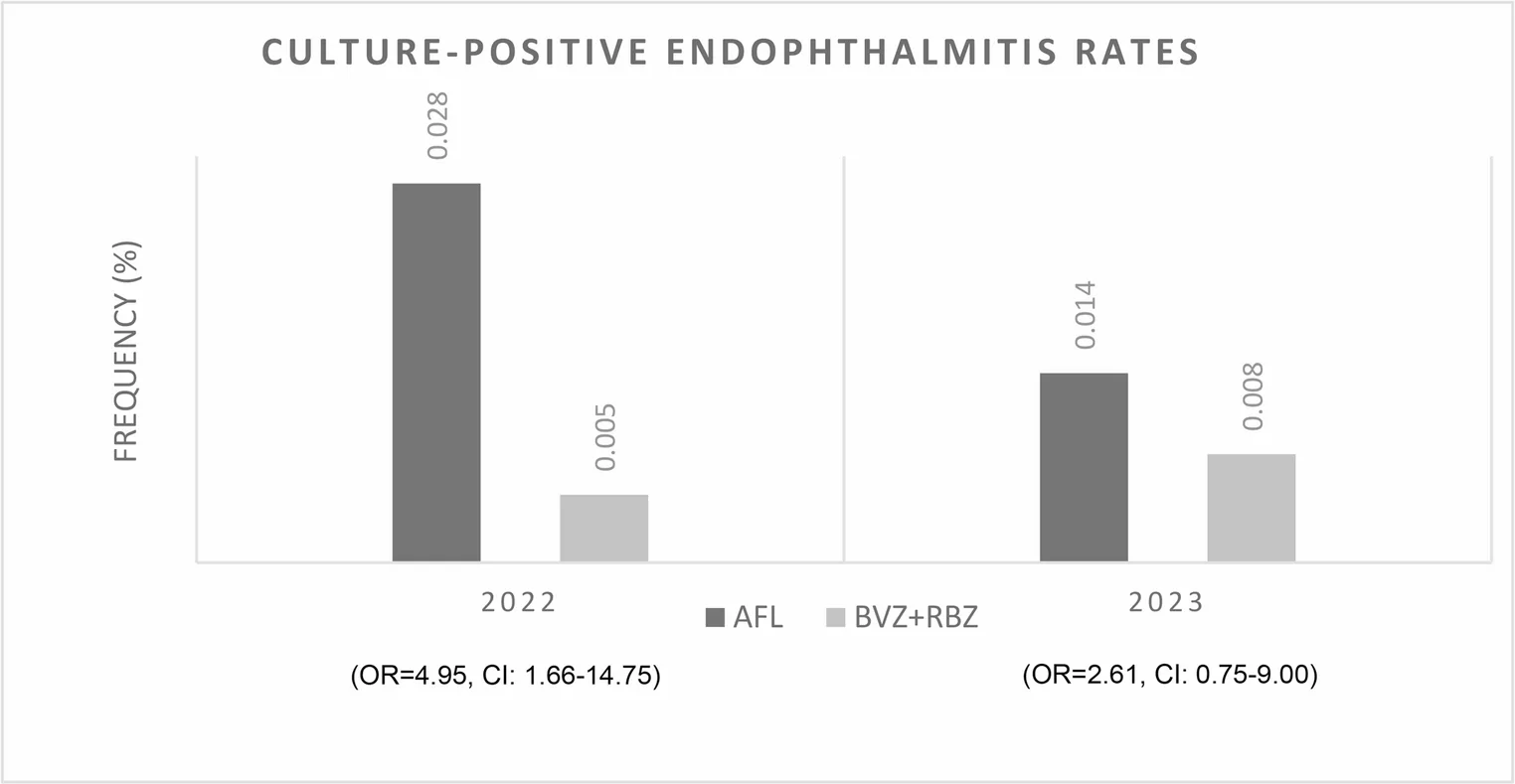
– comparison of culture-positive endophthalmitis rates with aflibercept compared with bevacizumab and ranibizumab in 2022 and 2023

Of the total (31) aflibercept-related endophthalmitis cases in 2022 and 2023, 14 (45.2%) were culture-positive, 14 (45.2%) culture-negative and 3 (9.6%) cases had no cultures obtained (clinical diagnosis). Among the 29 cases following bevacizumab or ranibizumab injections, 10 (34.5%) were culture-positive, 16 (55.1%) culture-negative, and 3 (10.4%) had no cultures obtained. Culture-positive endophthalmitis rates in 2022 were significantly higher following IVT-AFL injections (*N* = 9, 1:3554) compared with the combined rates of bevacizumab and ranibizumab (*N* = 5, 1:17,582), (OR = 4.95, 95% CI 1.66–14.75, *p* = 0.004). In 2023, the rates of culture-positive endophthalmitis following IVT-AFL (*N* = 5, 1:7117) were still higher, but not statistically significant, compared with the rates of bevacizumab and ranibizumab combined (*N* = 5, 1:18,543), (OR = 2.61, 95% CI 0.75-9.00, *p* = 0.13), (Fig. 2). The decrease in culture-positive endophthalmitis after the switch to PFS IVT-AFL was without statistical significance (*p* = 0.204).

*Culture-positive endophthalmitis rates were higher following intravitreal injection of aflibercept compared to bevacizumab and ranibizumab in 2022. In 2023*,* after transition to the pre-filled syringe formulation*,* rates remained numerically higher in the aflibercept group*,* but the difference was not statistically significant. AFL= aflibercept; BVZ = bevacizumab; RBZ = ranibizumab.*

Culture-negative endophthalmitis rates in 2022 following IVT-AFL were 1:6397 (*N* = 5), without statistically significant difference when compared with the rates of 1:7992 (*N* = 11) after bevacizumab and ranibizumab combined (OR = 1.32, 95% CI 0.46–3.81, *p* = 0.61). In 2023, the culture-negative rates of IVT-AFL − 1:3953 (*N* = 9) were significantly higher than that of bevacizumab and ranibizumab combined − 1:18,543 (*N* = 5) (OR = 4.69, 95% CI 1.57–14.02, *p* = 0.005). The culture-positive and culture-negative groups had no significant difference when compared by factors such as visual acuity at presentation, final visual acuity and days to intervention. Patients that underwent pars plana vitrectomy (PPV) had significantly higher proportion of culture- positive endophthalmitis cases (73.9%) compared with tap and inject (TAI) (22.6%) (OR = 9.71, 95% CI 2.77–34.08, *p* < 0.001). A significant difference was also noted when looking at aflibercept results only.

## Discussion

This multi-center, retrospective study examined endophthalmitis rates following intravitreal anti-VEGF injections across two years in ten different medical centers. Our findings reveal significantly higher endophthalmitis rates for aflibercept, compared to bevacizumab and ranibizumab, irrespective of syringe type. The rates of culture-positive endophthalmitis cases were reduced and were similar between aflibercept and other anti-VEGF agents, when using prefilled syringes in 2023.

The rate of culture-negative endophthalmitis in this study (45.2%) was similar to the rates described before in the literature [23, 26]. Corresponding to the discussion in these studies, it might be suggested that some of the endophthalmitis cases have been misdiagnosed and possibly had a sterile inflammatory reaction to the injected agent. As all cases in this study were diagnosed and treated as infectious endophthalmitis, it remains unclear whether some of these eyes had in fact a sterile inflammatory response rather than infectious endophthalmitis.

The transition from vial presentation to PFS for aflibercept in early 2023 did not result in a statistically significant change in overall endophthalmitis rates. Similar results were noted by Morioka et al. [25] that showed surprisingly low endophthalmitis rates following vial and PFS injections of aflibercept, but with no statistically significant difference between the two groups. The authors suggested that the lack of significance could be associated with the infrequent incidence of endophthalmitis in this study (1:10,835 for the vial and 1:21,842 for the PFS) requiring a larger sample size to show statistical significance.

Several hypotheses may explain the higher endophthalmitis rates observed with aflibercept. The differences in molecular structure or formulation between aflibercept and other anti-VEGF agents could influence inflammatory responses. Aflibercept is a larger fusion protein with a distinct molecular structure and high affinity for VEGF, compared to ranibizumab and bevacizumab [27, 28], which may theoretically influence its immunogenic potential. Prior studies have suggested that structural and formulation differences between anti-VEGF agents may affect their propensity to induce sterile intraocular inflammation [29]. While this theory might account for some of the misdiagnosed, culture-negative cases, current evidence does not demonstrate that molecular size itself increases the risk of infection. Therefore, any potential relationship between aflibercept’s molecular characteristics and the observed infection rates should be considered speculative. Another factor to consider is the difference in syringe design between the pre-filled aflibercept and other agents. Hinkle et al. [30] compared the design of aflibercept and ranibizumab pre-filled syringes and their effect on the amount of injected volume and possible risk of contamination. They found that even minimal changes in the syringe’s stopper position can have a significant effect on the amount of injected drug, with twice the volume/distance ratio using the pre-filled aflibercept syringe. The researchers also speculated that the aflibercept PFS design, with the stopper connected to the plunger, causes a retraction of the stopper when the plunger is released, leading to the aspiration of bacteria into the syringe, possibly increasing the risk of endophthalmitis. Lastly, while all participating centers used similar injection protocol, subtle variations in drug preparation or administration might have occurred.

The significantly lower rates of culture-positive endophthalmitis in patients that underwent TAI may reflect differences in sampling techniques rather than a true difference in the underlying infectious process. Issa et al. [31] also found a significant difference in the rate of culture negative endophthalmitis after TAI vs. PPV – 54.6% and 19.4%, respectively. This finding may be explained by several factors. First, the smaller intraocular sample typically obtained during TAI compared with the larger vitreous sample collected during PPV may reduce microbiological yield. Second, it is possible that some culture-negative cases represent sterile inflammatory reactions rather than true infectious endophthalmitis. At present, however, it is difficult to distinguish between these possibilities, and both differences in sampling methodology and the presence of sterile inflammation may contribute to the higher proportion of culture-negative cases observed after TAI. Of note, the default method of intervention for endophthalmitis is different in every medical center. Out of 10 centers, 6 prefer PPV as their primary intervention, three TAI, and one refers to other institutions for management.

We observed a notable, though not statistically significant, decrease in culture-positive endophthalmitis rates following the switch to PFS. This latter trend aligns with previous studies that have reported reduced culture-positive and total endophthalmitis rates with the adoption of PFS for other anti-VEGF agents [21, 32]. The lack of a significant reduction in overall endophthalmitis rates with PFS use for aflibercept contrasts with some previous findings for other anti-VEGF agents [19, 23]. This could indicate that the culture-negative cases might represent sterile inflammatory responses, as mentioned before. Alternatively, PFS could theoretically be more effective in preventing certain types of infections, particularly those caused by more virulent organisms that are more likely to yield positive cultures. Nevertheless, no evidence supporting this theory has been published so far, making further research necessary to determine its validity.

The multicenter design and large injection volume strengthen the reliability of our results. However, despite similar injection protocols, there could be unaccounted procedural and environmental variations across centers. Other limitations of our study include its retrospective nature and the relatively small number of endophthalmitis cases, especially in the culture-positive group, which might explain the lack of statistical significance.

In conclusion, the transition to prefilled syringes for aflibercept did not significantly reduce overall endophthalmitis rates but showed a trend towards decreased culture-positive infections. Understanding the reason for the significantly higher rates of post-injection endophthalmitis after aflibercept compared with other anti-VEGF agents is of importance and should be further investigated.

## Data Availability

No datasets were generated or analysed during the current study.
